# Reproduction of porcine ear necrosis (ear-tip necrosis) following intradermal inoculation of pigs with *Fusobacterium necrophorum*

**DOI:** 10.1371/journal.pone.0337536

**Published:** 2025-11-20

**Authors:** Matheus de O. Costa, Roman Nosach, Maite H. M. de Almeida

**Affiliations:** 1 Large Animal Clinical Sciences, Western College of Veterinary Medicine, University of Saskatchewan, Canada; 2 Population Health Sciences, Faculty of Veterinary Medicine, Utrecht University, Utrecht, The Netherlands; Sungkyunkwan University - Suwon Campus: Sungkyunkwan University - Natural Sciences Campus, KOREA, REPUBLIC OF

## Abstract

Porcine ear necrosis (PEN) (also referred to as ear-tip necrosis, ETN) is a syndrome of global presence and unclear aetiology. Initially reported in the 1950s, many different infectious and non-infectious causes have been suggested as the causative(s) agent(s), but none has been confirmed in controlled studies. Here, we investigated the aetiology of PEN using pure culture of bacteria associated with lesions in controlled animal trials. A commercial farm with no history of ear-tip necrosis was identified and used as the source for 5-week-old pigs. Two independent trials were initially executed with identical designs. Piglets (=12/trial) were intradermally inoculated with either pure cultures of *Staphylococcus hyicus* or *Fusobacterium necrophorum* (left ear, n = 10) or sterile media (right ear, n = 10). Two pigs in each trial were not inoculated, serving as sentinels. A third trial used *F. necrophorum* as the inoculum, 3 pigs as sentinels and 9 as inoculated. All animals were clinically monitored daily following challenge, and an ear score was used to follow disease progression. All ears inoculated with *S. hyicus* remained lesion free. Four out of ten and 7/9 pigs challenged with *F. necrophorum* developed lesions undistinguishable from PEN, including necrosis and loss of portions of the ear pinna (P < 0.001). *F. necrophorum* was isolated from 4/10 and 7/9 pigs that developed necrotic lesions. Histopathology after resolution of necrosis revealed granulomatous tissue. Evidence presented here suggests that *F. necrophorum* causes PEN-like lesions, as seen in commercial barns. It is therefore suggested as the etiological agent of this syndrome.

## Introduction

Swine ear-tip necrosis (ETN) or porcine ear necrosis, (PEN) is a disease of the ear pinna characterized by progressive necrosis of the integument and adjacent connective tissue in nursery and early-grower pigs. Initial lesions were suggested to be localized on the tip of the pinna, described as local erythema and edema, followed by necrosis and progressive loss of the pinna edges [1]. This disease has been reported in all of the major global pork-producing countries [2,3]. Depending on unknown factors, lesions may advance towards the base of the pinna, with consequent complete loss of the external ear in severe cases [1,4]. One study suggested that PEN may have limited to no effect on animal performance, with average daily gain not differing between lots with high incidence and low incidence of the disease [5]. Contrasting data recently evidenced that any degree of PEN during the nursery-grower phase had a significant impact growth rate [6]. Regardless of its production impact, it is a seemingly painful condition and an animal welfare concern with societal implications, as the public may perceive PEN as a proxy for poor animal husbandry and abuse. Finally, ears with open wounds are a port-of-entry for opportunistic pathogens that may lead to local or generalized infection – secondary issues to PEN that can adversely affect weight gain and animal performance, and potentially food safety if associated with carcass abscessation.

Since its description over 70 years ago, PEN has been suggested to be a multifactorial issue linked, at least partially, to the environment (type of flooring, air quality, humidity), animal behaviour (ear-chewing) and site management (high stocking-density, [3,7]. Uncontrolled, field trials suggested the involvement of mycotoxins, *Staphylococcus hyicus*, or porcine circovirus type 2 (PCV-2) in the aetiology of PEN [1,4,8,9]. To date, inconsistent evidence has been found of PCV-2 or mycotoxins being linked to vasculopathy in ear sections of affected pigs [4,10,11]. In parallel, one study explored the aetiology of PEN under controlled conditions with a defined challenge agent. Researchers used *Treponema pedis*, a spirochete detected in lesioned ears, to produce an inoculum and directly deliver it into the ear following a week of ear bandaging to increase skin moisture [8]. No lesions were identified up to 56 days following inoculation. More recently, two independent studies used DNA sequencing to profile the microbiome associated with PEN lesions [12,13]. While no conclusive evidence was reported regarding the etiology of PEN, both concluded that infectious agents played a role in lesion development. Currently, two pathophysiological mechanisms have been postulated to explain the development of lesions: i) PEN results from skin trauma followed by intradermal introduction of the agent(s) responsible for causing disease, or ii) PEN develops following systemic dissemination of the agent(s), resulting in local vasculopathy that progresses to ischemia and eventually necrosis of the pinna [1,5,13].

Here we hypothesized that PEN is a disease of infectious etiology that is experimentally reproducible when susceptible animals are exposed to the agent(s). Using the challenge strategy developed by Costa, Nosach (12), our objective was to induce PEN in a susceptible population of pigs using pure culture of *Staphylococcus hyicus* or *Fusobacterium necrophorum* as inocula.

## Materials and methods

This project was approved by the University of Saskatchewan’s Animal Research Ethics Board and adhered to the Canadian Council on Animal Care guidelines for humane animal use (permit #20190087).

### Animal trials

**Fig 1:**
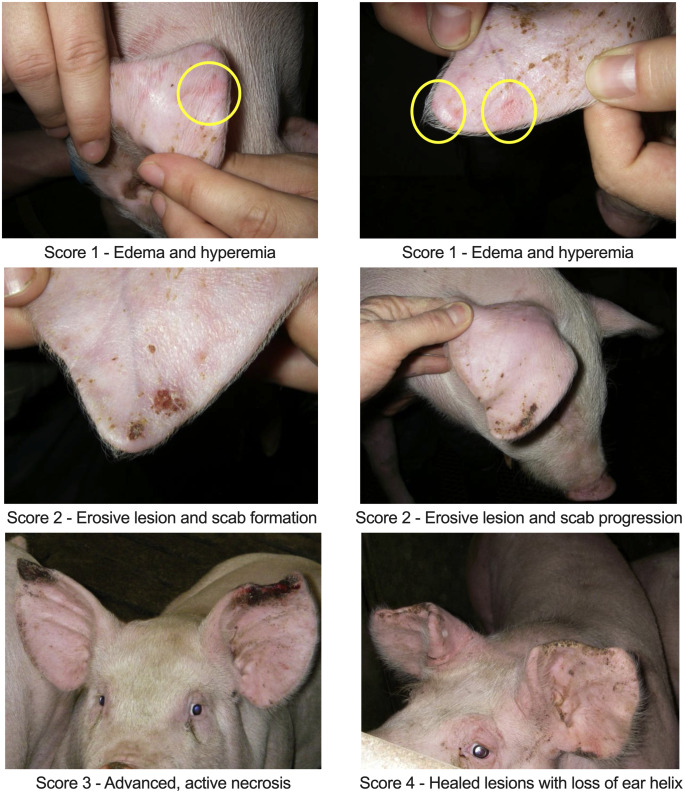
Ear tip necrosis lesion scoring chart. Photos obtained during a clinical outbreak.

Three randomized, single-blinded independent trials were executed using a previously described inoculation strategy and identical designs – they differed only on the inoculum used [12]. Animals were sourced from a highly biosecure, commercial farm with no history of PEN and free of major swine pathogens including porcine reproductive and respiratory virus (PRRSV), influenza A virus of swine (IAV‐S), *Mycoplasma hyopneumoniae*, *Erysipelothrix rhusiopathiae, Salmonella enterica* serovar Cholerasuis, *Actinobacillus suis, Actinobacillus pleuropneumoniae, Lawsonia intracellularis*, *Brachyspira hyodysenteriae* and *B. hampsonii*. Pigs were vaccinated for PCV-2 using a commercial vaccine. In each trial, healthy 5-weeks old crossbred piglets (mean body weight 6.1 kg), free of PEN-like clinical signs (n = 12) were transferred from the source farm and acclimatized in a biocontainment level 2 facility for 7 days. At arrival, pigs were blocked by weight (to avoid fights and improve welfare) and sorted into 2 pens for trials 1 and 2 (2.4 m x 3.6 m, 0.7 m^2^/pig) within the same room, with 6 pigs/pen. For trial 3, pigs were divided into 3 pens (2 m x 1.4 m), at the same stocking density, with 4 pigs/pen. Room temperature was set to 27°C and reduced by 1°C/week, and heat lamps were provided upon arrival. Pigs in each pen were randomly allocated into two groups: sentinel (n = 1) or inoculated (n = 5). In the inoculated cohort, the right ears were assigned as controls (n = 10, sham inoculum comprising sterile brain-heart infusion, BHI), while the left ears were inoculated (n = 10, pure culture of live bacterial inoculum). To monitor and account for the spontaneous occurrence of PEN, sentinel pigs (n = 2 trials 1 and 3, n = 3 for trial 3), which were not subjected to inoculation, were employed in the study. All animals in all trials had no visible PEN lesions through the acclimation period. Starting on 0 days post-inoculation (dpi), pigs were monitored by veterinarians blinded to group identity twice daily for any clinical abnormalities for up to 21 days or until lesions healed. This included body condition, skin color, responsiveness, rectal temperature, and ear lesions (S1 Table). Ear lesions were scored as: 0) no visual abnormalities; 1) edema and hyperemia visible, initial lesions, no epithelial erosion or ulceration; 2) the initial swelling erodes through the skin surface to form a scab; 3) necrotic tissue is present; 4) healed lesions with loss of ear tissue (Fig 1). Scoring was performed by a subject blinded to the inoculum identity. Animals were euthanized if animal welfare was a concern (score >3 in one or more clinical assessment categories), or if ear lesions were not progressing in severity. Euthanasia was performed by captive bolt followed by exsanguination, and ear samples were fixed in 10% buffered formalin. Control and inoculated pinnae were sectioned on the coronal plane and swabs used to sample the subcutaneous tissue and used for bacterial culture.

### Bacterial culture and inoculation

Trial 1 used as inoculum a *Staphylococcus hyicus* strain obtained from the ear of a pig with PEN [12]. The strain was isolated in mannitol salt agar (Oxoid, Ottawa, Canada) at 37°C. The isolate was prepared for inoculation by culturing it to log phase in BHI broth, at 37°C, for 16 hours, under aerobic conditions, to a final load of 10^8^ CFU/mL.

Trial 2 and 3 inoculum used *Fusobacterium necrophorum* subspecies *necrophorum* (biotype A) isolated from the ear of a pig with PEN [12]. The isolate was first cultured in 5% sheep blood agar (BD, Mississauga, ON, Canada) anaerobically at 37°C for 48 hours, and then propagated in pre-reduced BHI broth, at 37°C, anaerobically, for 42 hours until OD_600_ of 1.1 was reached, totalling 2.3 x10^6^ CFU/mL for Trial 2, and an OD_600_ of 1.3 and 1.7 x10^7^ CFU/mL for Trial 3. Due to the filamentous growth of *F. necrophorum,* this is likely an underestimation of the total number bacterial of cells inoculated. Anaerobiosis was generated using a commercial system (Anaerogen, Oxoid Limited, Basingstoke, United Kingdom). Subcutaneous swabs from Trial 2 and 3 were cultured in pre-reduced 5% sheep blood columbia agar (BD, Mississauga, ON, Canada) anaerobically for up to 120 hours, or until β haemolysis was observed. β-hemolytic isolates were profiled by Gram-staining and then identified using matrix-assisted laser desorption/ionization time-of-flight (MALDI-TOF) mass spectrometer.

Prior to and immediately following inoculation, aliquots of the inocula (both control and inoculated) were cultured in the appropriate media to verify pathogen viability. Inoculation was performed as previously described [12], with the challenged ear side (right or left) randomly chosen using the rand() function on Excel (V16, Microsoft Corporation, Redmond, USA). Piglets were manually restrained, as if for vaccination, and a commercially available needle-free injector system was used to introduce the inocula intradermally (Pulse 250, Pulse NFS, Lenexa, KS, USA). All control ears were inoculated first, followed by the inoculated ears. Each ear received 1 mL of a given inoculum once, on the rostral aspect of the pinna, 0.5 cm from the apex of the pinna, to simulate oral manipulation by other pigs.

At necropsy, all ears were sectioned at inoculation site (apex of the pinnae), or where lesions were visible, and samples were collected for histopathology (see method below) and bacteriology. Samples were plated on pre-reduced Columbia blood agar and incubated for 96 hours, or until colonies were visible. β-hemolytic isolates were profiled by Gram-staining and then identified using matrix-assisted laser desorption/ionization time-of-flight (MALDI-TOF) mass spectrometer.

### Staphylococcal exfoliative toxin PCR

The *S. hyicus* strain used was evaluated for harbouring the exfoliative toxin *shet*A gene using the following primer set: sheta_F 5’- GTATAGCGATTGCGGGATTG – 3’, sheta_R 5’- AGCCAAAACATAGGCTGCTG– 3’. Each reaction included and 2 μL of template DNA, in a final volume of 25 μL. PCR reactions were run on a plate containing a no-template control. All reactions were performed in duplicate. Thermocycling parameters included an initial denaturation (95°C for 3 min.), followed by 40 cycles of 95°C for 15 sec., 63°C for 15 sec., 72°C for 15 sec., and a final extension at 72°C for 5 min. A dissociation curve was subsequently performed for 81 cycles at 0.5°C increments from 55°C to 95°C. Fluorescent signals were measured every cycle at the end of the annealing step and continuously during the dissociation curve data collection. All resulting data was analyzed using iQ5 Optical System Software (Bio-Rad Laboratories (Canada) Ltd., Mississauga, ON).

### Histopathology analysis

Formalin fixed ear samples collected at euthanasia were embedded in paraffin for staining with haematoxylin and eosin (H&E) and Warthin-Faulker stains.

### Statistical analyses

For trials 2 and 3, ear lesion scores between sides (right vs left) were compared using Wilcoxon signed-rank test, and disease incidence (control vs inoculated ears) was analyzed using Fisher’s exact test.. Statistical analyses were performed using SPSS (v21, IBM, Illinois).

## Results

### Trial 1 – S. hyicus inoculum

**Fig 2:**
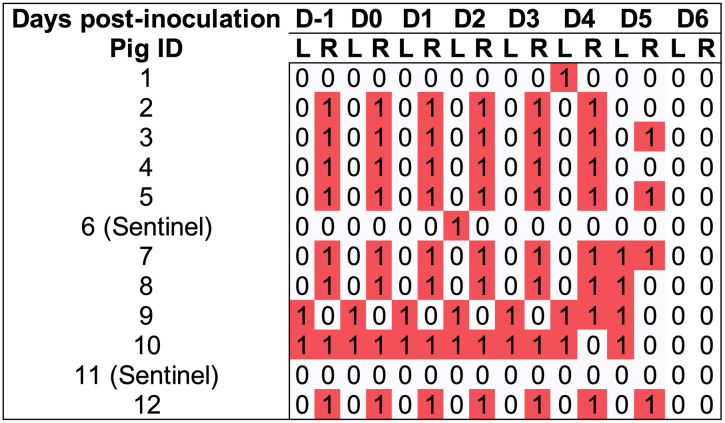
Heatmap of (Right and Left) ear necrosis scores throughout the experimental period of trial 1, using *Staphylococcus hyicus* as the inoculum.

The *S. hyicus* strain was found positive for the *she*tA by PCR (data not shown). Growth was observed in agar plates used to test inoculum viability, whereas the sham inoculum did not result in any growth. All animals were free of PEN-like lesions prior to inoculation. Control (right) and inoculated (left) ears from all pigs (including sentinels) remained free from lesions, other than erythema following inoculation, throughout the studied period (Fig 2). By 6 dpi, all ears were free of lesions (ear lesion score 0). As there were no visible alterations to the pinnae, animals were euthanized. No other clinical signs were observed during the trial (S1 Table). Given the lack of clinical signs and gross lesions, histopathology was not performed.

### Trial 2 – F. necrophorum inoculum

**Fig 3:**
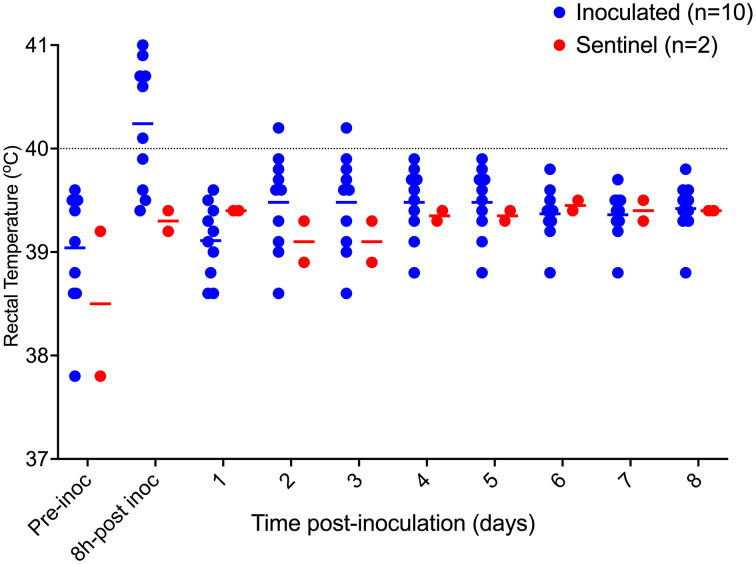
Post-inoculation rectal temperature from pigs inoculated (n = 10) with *F. necrophorum* and sentinels (n = 2) in Trial 2. Horizontal bars depict group average, dotted line shows the fever threshold for pigs at this age.

**Fig 4:**
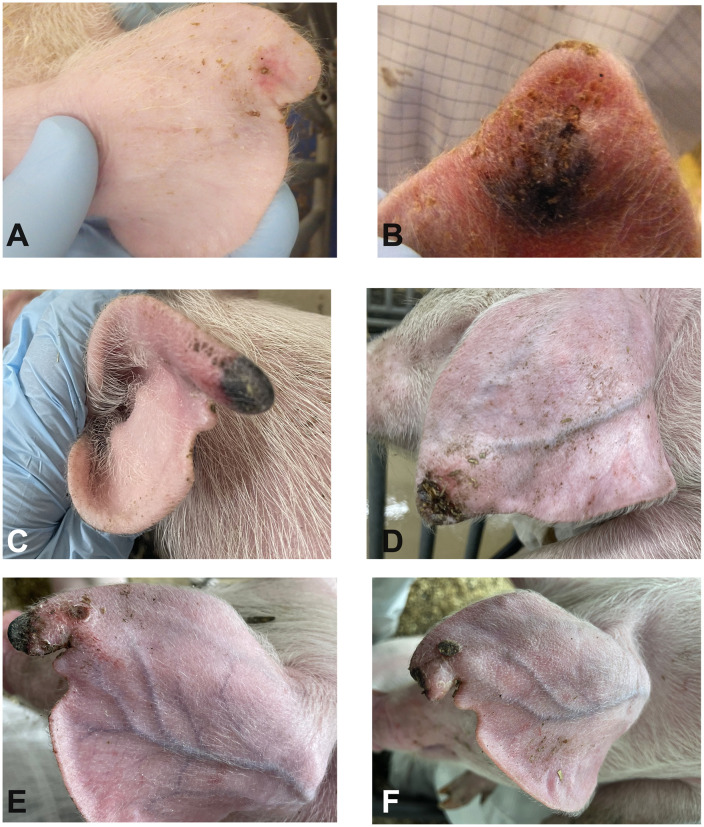
Representative photos depicting lesion progression from Trial 2 (*F. necrophorum*). **A**- Control ear sham inoculated, 8 hours post-inoculation. **B**- Inoculated ear 24 hours post-inoculation showing subcutaneous haemorrhage. **C-** Inoculated ear 2 days post-inoculation showing haemorrhage and early necrosis. **D and E-** Inoculated ears 4 days post-inoculation, showing progressive tissue dehydration and necrosis. **F-** Inoculate ear 6 days post-inoculation with tissue loss, sloughed necrotic tissue.

**Fig 5:**
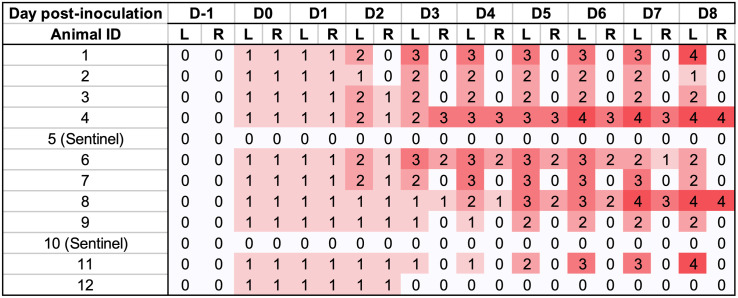
Heatmap of (Right and Left) ear necrosis scores throughout the experimental period of Trial 2, using *Fusobacterium necrophorum* as the inoculum delivered to the left ear of inoculated pigs (n = 10). Score 0) no visual abnormalities; 1) edema and hyperemia visible, initial lesions, no epithelial erosion or ulceration; 2) the initial swelling erodes through the skin surface to form a scab; 3) necrotic tissue is present; 4) healed lesions with loss of ear tissue.

Pure culture with the expected phenotypical characteristics were observed in blood agar plates used to test inoculum viability immediately following inoculation, whereas sham inoculum did not result in any growth. Pigs were inoculated with *F. necrophorum* on the left ear, and the right ear was sham inoculated. At eight-hours post-inoculation the mean rectal temperature of inoculated pigs was 40.2°C ± 0.6°C, and sentinels was 39.3°C ± 0.1°C (Fig 3). Rectal temperature from all pigs remained within normal limits for the rest of the studied period. Within the first 8 hours post-inoculation, control ears developed hyperemia around the inoculation site (Fig 4A). Ears receiving *F. necrophorum* progressed from hyperemic (Fig 4A) to haemorrhagic within 8 hours post-inoculation (Fig 4B). By 1 dpi, hyperemic areas expanded and subcutaneous haemorrhage was observed in 3/10 of the inoculated pigs (Fig 4B). From 2–6 dpi, lesions did not expand in surface area, but affected sites of the pinnae progressively became dehydrated and devitalized (starting from the most distal point) in 4/10 (ear score 4) of the pigs (Fig 4C, 4D, 4E). As early as 6 dpi, necrotic tissue sloughed off the pinnae from affected animals (Fig 4F). By 8 dpi, lesions were no longer progressing in area or severity, and animals were euthanized. A summary of ear lesion scores is shown in Fig 5, evidencing lesion progression in 4/10 animals that reached a score 4 (necrosis) by day 4–6. Interestingly, in 2/4 inoculated pigs (#4 and #8) that developed necrosis also had the contralateral control ear (right) develop lesions. Sentinels remained lesion-free throughout the study period. No other clinical signs were observed (S2 Table). A significant difference between left vs right ear scores of the inoculated group was found (P < 0.001), whereas disease incidence was not different between control and inoculated groups (P = 0.6)

**Fig 6:**
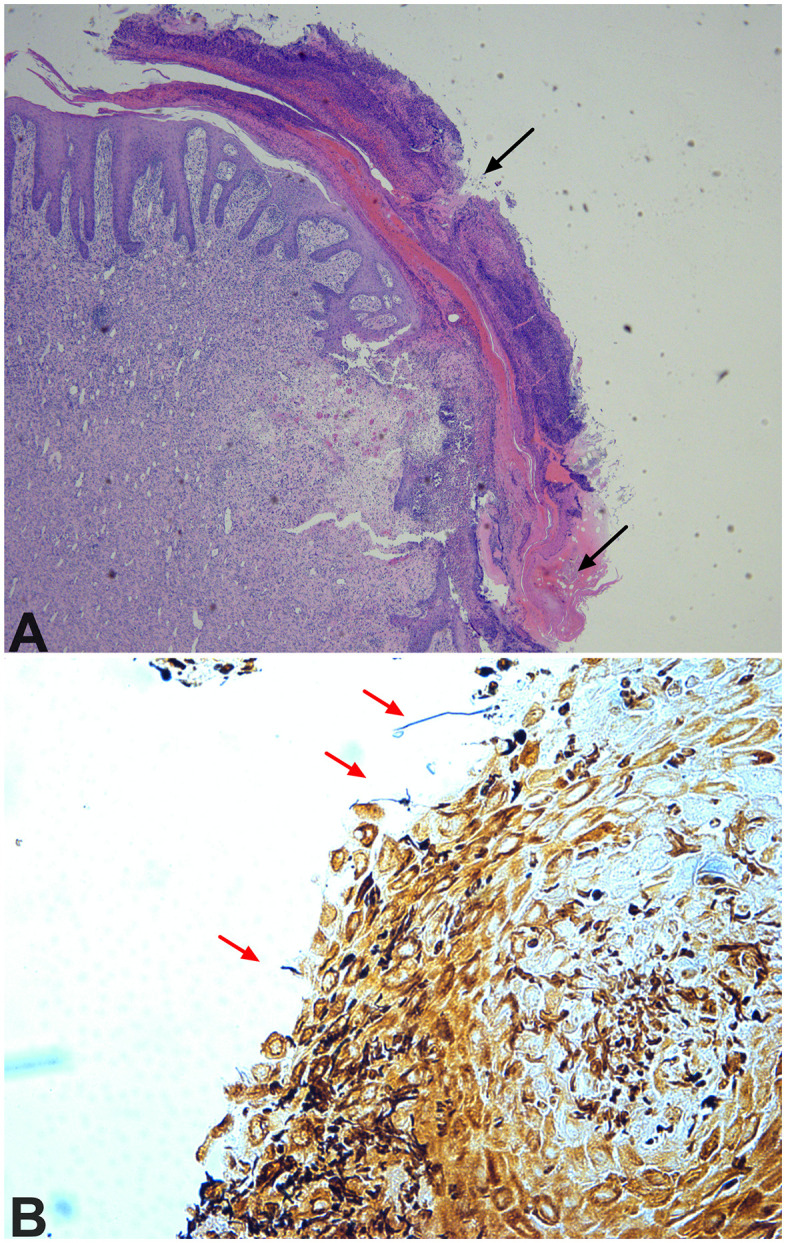
Histopathology from ear samples collected post-mortem from pigs in Trial 2 (*F. necrophorum*). **A-** Representative H&E stained section of the ear from a pig that developed necrosis and lost a section of the ear. Loss of epithelial coverage and ulceration is observed (black arrows), as well as granulation tissue. **B-** Higher magnification within the same section stained with Warthin-Faulkner revealed filamentous structures within and around the ulcerated sections, suggestive of *F. necrophorum* (red arrows).

*F. necrophorum* was isolated and identified on samples from the left ears of 4/10 (#1, #4, #8 and #11), and right ears of 2/10 (#4 and #8) inoculated pigs, and none of the sentinel pigs. Given that lesions were allowed to heal so clinical progression could be observed, microscopical examination of pinnae sections from inoculated pigs was mostly characterized by marked granulation tissue. The overlying epidermis ranged from markedly hyperplastic with prominent *rete* ridges to ulcerated and covered by a thick crust composed of parakeratosis admixed with hemorrhage, fibrin, numerous neutrophils, nuclear debris and micro-colonies of 1–2 µm Gram-positive cocci (Fig 6A). Vasculitis or thrombosis were not present. Filamentous structures, suggestive of *Fusobacterium* spp., were also visible on lesioned surfaces in silver-stained sections (Fig 6B).

### Trial 2 – F. necrophorum inoculum confirmation

**Fig 7:**
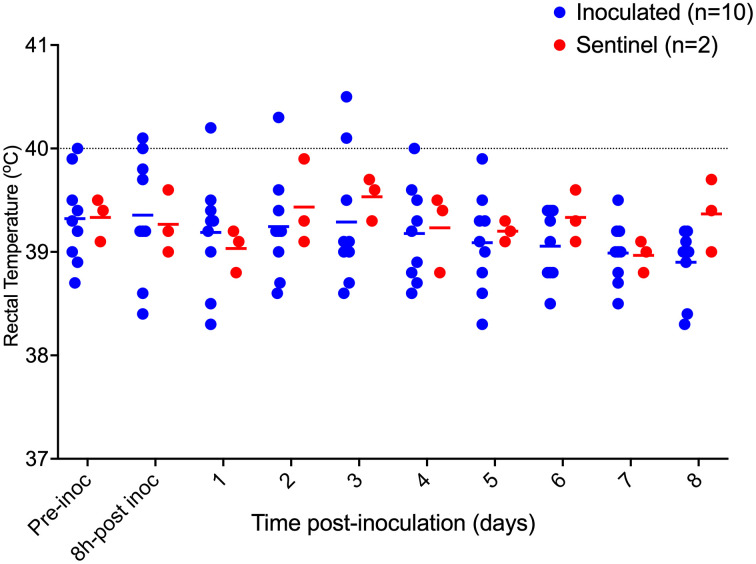
Post-inoculation rectal temperature from pigs inoculated (n = 9) with *F. necrophorum* and sentinel pigs (n = 3) from Trial 3. Horizontal bars show group average for a given sampling point, dotted line represents the fever threshold for nursery pigs.

**Fig 8:**
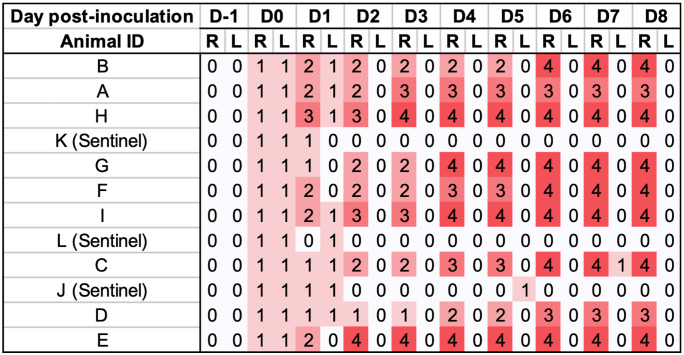
Heatmap of (Right and Left) ear scores during the experimental period of Trial 3, with *Fusobacterium necrophorum* inoculated to the right ear of inoculated pigs (n = 9). Score: 0) no visual abnormalities; 1) edema and hyperemia visible, initial lesions, no epithelial erosion or ulceration; 2) the initial swelling erodes through the skin surface to form a scab; 3) necrotic tissue is present; 4) healed lesions with loss of ear tissue.

**Fig 9:**
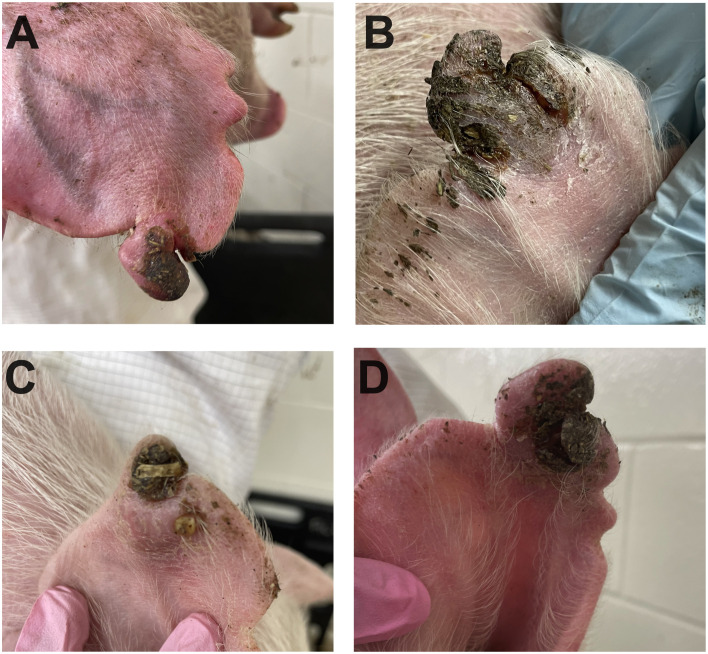
Representative lesions of porcine ear necrosis identified in Trial 3, using *F. necrophorum* as inoculum. **A-** Inoculated ear 3 days post challenge depicting a necrotic area with inflamed borders where viable tissue is still present. **B, C and D-** Inoculated ear 5 and 6 days post-challenge, with necrotic tissue partially separated from viable tissue.

On −5 dpi, pig E was treated once with ceftiofur intramuscularly, per label (Excede, Zoetis, Kirkland, Canada) due to increased rectal temperature, reduced feed intake and lethargy. Clinical signs resolved following this treatment. Colonies showing the expected phenotypicial characteristics for *F. necrophorum* were recovered from the inocula used. Sham inocula resulted in no growth. Inoculated pigs received *F. necrophorum* in their left ears, whereas the right ears were sham inoculated. At 8 hours post- challenge, the average rectal temperature for inoculated pigs was 39.3°C ± 0.6°C, and sentinels was 39.1°C ± 0.3°C. Pig A had > 40°C rectal temperature on 1 and 2 dpi, and E on 0 and 3 dpi (Fig 7). Similarly to Trial 2, all ears from inoculated pig developed hyperemia on the site of inoculation by 8 post-inoculation. Lesion progression followed a similar pattern as presented in Trial 2. Necrosis (score 4) was identified as early as 3 dpi in 1 pig (H), and affected 7/10 inoculated right ears with no counter-lateral disease detected at any time point. A summary of ear lesion scores for this trial is shown in Fig 8, showing lesion progression to necrosis was achieved as early as 3 dpi. Sentinels remained free of lesions throughout the trial. Ear scores were significantly different between left and right ears of inoculated pigs (P < 0.001), and disease incidence between inoculated and sentinel pigs was also statistically significant (P = 0.045). Representative lesions identified in this trials are shown in Fig 9.

*F. necrophorum* was isolated identified on terminal ear swabs from the right ears of 7/9 (B, H, G, F, I, C and E) pigs, and none of the left ears or any samples from sentinel pigs. Histopathological findings from the right ears of inoculated pigs were similar to those identified in Trial 2.

## Discussion

To the best of our knowledge this is the first replication of a syndrome undistinguishable from PEN using a single, defined agent under controlled conditions. Until now it was only suggested that the aetiology of PEN was, at least partially, infectious [12]. The previously published model was unable to induce necrosis, whereas here we used a single intradermal inoculation of *F. necrophorum* to induce mild-to-moderate lesions undistinguishable from PEN in clinically healthy pigs *m*, and the bacterium was recovered from lesioned sites. This finding suggests that PEN has a bacterial aetiology, and that brakeage of the pinna skin barrier results in clinical signs.

The etiology of PEN has been postulated for decades. *Treponema pedis*, *Staphylococcus aureus*, *Staphylococcus hyicus*, PCV-2, porcine respiratory and reproductive syndrome virus (PRRSV), all have been suggested as etiological agents of this disease [1,3,4,8,10,11]. Multiple mycotoxins (acetyldeoxynivalenol, diacetoxyscirpenol, ergosine, ergotamine, ergocornine, ergocryptine, ergocristine, deoxynivalenol, nivalenol, zearalenone, T2 toxin and HT2 toxin) have also been postulated to play a role in the development of PEN due to their vasotoxic properties [4]. These previous reports were based on clinical observations in a production setting. In only one instance a controlled clinical trial used a single, defined agent (*T. pedis*) to challenge pigs in an attempt to replicate the disease [8]. In this case, despite bandaging and scarification of the pinna skin, no lesions were observed.

*F. necrophorum* is a Gram-negative, non-spore forming, pleiomorphic strict anaerobe, previously known as the causative agent of hepatic abscess, foot rot, necrotic laryngitis and summer mastitis in ruminants [14]. It also causes mandibular abscesses in antelopes and marsupials, Lemierre’s syndrome and soft tissue abscesses in humans [15]. *F. necrophorum* is known to be part of the gastrointestinal tract microbiome of healthy pigs (including tonsils and soft palate) [16–19], suggesting it is ubiquitous in modern swine operations, which corroborates the world-wide PEN distribution. The pathogen has been suggested as the aetiological agent of hoof necrotic lesions [20], abscesses [21], proposed as a co-factor for the development of swine dysentery [22], endocarditis [23], face and neck edema, abscesses and polyarthritis [24], and necrotising stomatitis [25,26]. Interestingly, all of the syndromes cited above included necrotizing lesions as part of their clinical, gross or microscopical findings.

Recently, three independent studies using high-throughput DNA sequencing found *Fusobacterium* spp. as part of the microbiome within PEN lesions sampled from pigs in commercial farms [12,13,27]. Noteworthy, *Fusobacterium* spp. were the most prevalent [13] and most abundant [12] bacteria detected in those samples, as well as the one out of two taxa found only in affected ears [27]. In hindsight, both studies provided valuable evidence of the key role played by *F. necrophorum* in the pathogenesis of PEN. This bacterium possesses multiple virulence factors, including a leukotoxin, endotoxin, haemolysin, haemagglutinin, adhesins, dermonecrotic toxin, and proteases [15]. Following artificial brakeage of the pinna dermal barrier, this combination of virulence factors seem to provide *F. necrophorum* the tools needed to induce lesions undistinguishable from PEN. Increased capsule production may help the pathogen avoid the immune system while activating platelets [28,29]. The lack *F. necrophorum* leukotoxin toxicity towards swine leukocytes may help explain why lesions are limited to the site of injection, and sepsis was not observed as a consequence of inoculation [30]. This also helps explain while following inoculation only a transient, mild fever was observed in a few animals in Trials 2 and 3. Haemagglutinin produced by *F. necrophorum* leads to thrombi formation, initially in venules followed by arterioles [31]. Together with endotoxin, these proteins may play a role on intravascular coagulation that ultimately leads to the microscopic and gross lesions observed in PEN [32]. Previous authors have suggested that PEN may be a result of *F. necrophorum* invasion at a different site than the ear, succeeded by systemic infection that culminates with local endothelial damage and ischemia in the pinnae [3,4]. At this point, we believe this not to be the correct route of invasion. The main site colonized by *F. necrophorum* in healthy pigs is the gastrointestinal tract, as *F. necrophorum* is part of the gastrointestinal microbiota. However, translocation through the intestinal mucosa would require intestinal barrier failure, which is plausible (and expected) at weaning (4 weeks of age),but not at 9 weeks of age when initial PEN lesions are identified in the field [13]. While we recognize the bias of our inoculation strategy, our study supports the “outside-in” hypothesis that PEN follows dermal inoculation, such as oral manipulation by other pigs -in fact this has been associated with increased PEN prevalence [27]. However, we did not attempt any other inoculation strategy. Regardless of the invasion route, ischemia and necrosis are the outcomes observed with PEN in the field and in this study. Interestingly, in 2/4 pigs from Trial 2 *F. necrophorum* was able to disseminate systemically and reach the opposite ear, but none in Trial 3. We hypothesize that this may have been an effect of direct inoculum injection. It is plausible that bacterial emboli were inadvertently introduced in capillaries, gaining systemic circulation access and thus spreading to the counterlateral year even though the primary *port d’entrée* was the damaged skin. This must be further explored.

Intraepidermal abscesses, acanthosis, and hyperkeratosis were previously identified as microscopical lesions associated with active PEN [1,7]. In chronic cases, where the ear helix is lost, the remaining tissue was replaced by granulomae [4]. In this study, we chose to allow the full clinical progression of the disease prior to euthanizing animals. Therefore, at the time of sampling for histopathology, lesions had healed and only granulomatous tissue was present, as previously reported in chronic cases. Future studies looking at the samples collected at different disease stages can contribute to further our understanding of the disease.

*S. hyicus* is associated with exudative epidermitis, or greasy pig disease. It has been found live in PEN lesions in previous studies [1,3,9] and has gathered consistent support as (one of) the agents of PEN. The *Staphylococcus hyicus* strain used in this study harboured the gene *shet*A (codes for an exfoliative toxin suggested to play a role in the pathogenesis of exudative epidermitis) and was isolated from a PEN lesion [12,33]. Despite this, we did not observe any lesions following challenge using pure cultures of *S. hyicus*. Although it did not induce lesions alone, we cannot discard the potential role of *S. hyicus* in perpetuating or increasing the severity of PEN lesions. It may be that *F. necrophorum* alone is a necessary, but insufficient factor to induce severe PEN, and that a polymicrobial infection (alongside *S. hyicus*, for example) is required for complete ear loss.

In this study we were unable to induce complete ear pinna loss following challenge with *F. necrophorum*, as seen in severe cases in commercial farms. Also, tissue loss was observed in 4/10 of the challenged pigs in Trial 2, and 7/9 in Trial 3. This is likely a result of (or combination of) inadequate inoculum load, frequency of inoculation (as ear chewing is a repetitive behaviour), individual susceptibility levels (passive or active antibodies). We hypothesize that repeated inoculation of *F. necrophorum* into the lesioned sites, to simulate ear biting, may be required to ensure lesion area progression. Ear biting was recently shown to be a key factor leading to the increase incidence of PEN [34]. A second hypothesis is that other microbe(s), such as *S. hyicus*, may be required for increased lesion area. Further investigation is needed to clarify this limitation, and to help improve the model and our understanding of PEN. We also expect that *F. necrophorum* may be linked to other necrotic syndromes in pigs, such as tail necrosis or swine inflammation and necrosis syndrome.

## Conclusions

Here we presented initial evidence that intradermal inoculation of healthy pig ears with *F. necrophorum* results in a syndrome undistinguishable from PEN. These results support the causal relationship between this bacterium and necrosis of the ear in swine. The global relevance and welfare challenges linked to PEN make the work described here pivotal towards the control and, perhaps eradication of this disease. Research is on-going in our laboratory to develop tools to prevent and treat PEN more efficiently, and without the need for antimicrobials.

## Supporting information

S1 Table Trial 1–clinical monitoring scores. (PDF)

S2 Table Trial 2–clinical monitoring scores. (PDF)

S3 Table Trial 3–clinical monitoring scores. (PDF)
